# Longitudinal profiling of the burn patient cutaneous and gastrointestinal microbiota: a pilot study

**DOI:** 10.1038/s41598-021-89822-z

**Published:** 2021-05-21

**Authors:** Kelly M. Lima, Ryan R. Davis, Stephenie Y. Liu, David G. Greenhalgh, Nam K. Tran

**Affiliations:** 1 Department of Pathology and Laboratory Medicine, University of California Davis, 4400 V St., Sacramento, CA 95817 USA; 2 Division of Burn Surgery, Department of Surgery, 2221 Stockton Blvd., Sacramento, CA 95817 USA

**Keywords:** Clinical microbiology, Molecular medicine, Microbiology techniques

## Abstract

Sepsis is a leading cause of morbidity and mortality in patients that have sustained a severe burn injury. Early detection and treatment of infections improves outcomes and understanding changes in the host microbiome following injury and during treatment may aid in burn care. The loss of functional barriers, systemic inflammation, and commensal community perturbations all contribute to a burn patient’s increased risk of infection. We sampled 10 burn patients to evaluate cutaneous microbial populations on the burn wound and corresponding spared skin on days 0, 3, 7, 14, 21, and 28 post-intensive care unit admission. In addition, skin samples were paired with perianal and rectal locations to evaluate changes in the burn patient gut microbiome following injury and treatment. We found significant (*P* = 0.011) reduction in alpha diversity on the burn wound compared to spared skin throughout the sampling period as well as reduction in common skin commensal bacteria such as *Propionibacterium acnes* and *Staphylococcus epidermitis*. Compared to healthy volunteers (*n* = 18), the burn patient spared skin also exhibited a significant reduction in alpha diversity (*P* = 0.001). Treatments such as systemic or topical antibiotic administration, skin grafting, and nutritional formulations also impact diversity and community composition at the sampling locations. When evaluating each subject individually, an increase in relative abundance of taxa isolated clinically by bacterial culture could be seen in 5/9 infections detected among the burn patient cohort.

## Introduction

Thermal injuries are prevalent worldwide, affecting over 8 million people globally in 2017^1^. Notwithstanding the prevalence, survival from thermal injury has been improving with advances in care. Data from the Global Burden of Disease 2017 study show a 46.6% (− 49.7 to − 38.8%) decrease in age-standardized mortality from 1990 to 2017^1^. Despite improved outcomes, significant predictors of mortality include burn size, age, sex, inhalation injury, and infection^2^. Burn patients are at increased risk of developing sepsis from infection, which is the most common cause of morbidity in this population^3^. The infection risk is due to many factors including: hypermetabolic response to injury, systemic inflammation, immune dysfunction, epidermal barrier disruption, and loss of commensal bacterial protection^4,5^. Early detection and treatment of sepsis reduces morbidity and mortality, subsequently, burn patients are closely monitored for signs of infection using clinical guidelines^3,6^. Due to the unique variability in types and severity of burn injury, algorithms are in development to monitor predictors of sepsis^7^.

Although early sepsis detection is crucial for appropriate treatment, clinical indicators only present once infection has led to a systemic response in the patient. Prevention of pathogenic microbial colonization is the primary tool employed in burn care. Topical and systemic antimicrobials are an integral part of sepsis prevention; however, commensal and pathogenic bacteria persist on the human skin and gut (two potential sites of bacterial seeding) despite use of antimicrobials. The mechanisms by which these populations experience a change in composition and interact with the host during disease is continually being discovered. Few studies have been conducted evaluating microbial composition change during treatment and recovery following severe thermal injury^8^. Immediate perturbations of cutaneous and gut populations after injury have not been explored. Currently, culture-independent methodologies by sequencing of the 16S ribosomal RNA gene or whole genome shotgun metagenomics are standard for microbiome assessment^9,10^. However, culture-independent microbial research of the microbiome is limited in the burn population.

A knowledge gap exists between recognizing infection, judicial antimicrobial use, and understanding complex host-commensal microbial interactions following thermal injury. To this end, understanding microbial population dynamics over the course of treatment and early detection of infection is imperative for sepsis management in this at-risk population. *There are three objectives for this study: (1) to provide preliminary data on longitudinal sampling of skin and gut microbiome in severely burned patients, (2) to understand changes in bacterial population dynamics over time and (3) to determine if culture-independent monitoring of these populations can predict bacterial overgrowth leading to infection or sepsis.* We hypothesize there will be a change in the community composition of the bacterial microbiota on burn patient’s skin and gut following severe burn injury and during treatment.

## Methods

### Selection of participants

#### Burn patients

All study protocols were reviewed and approved by the University of California (UC) Davis Institutional Review Board (IRB). Burn subjects were male or female adults (> 18 years old) enrolled on admission to the UC Davis Health (UCDH) burn intensive care unit (BICU) for treatment of thermal injury affecting ≥ 10% of the total body surface area (TBSA). Inclusion criteria required that the subject have burned and spared skin sites that are bilaterally symmetrical to each other with a spared site > 10 cm from affected tissue. Informed consent was obtained from the subject or a legally authorized representative in accordance with the UC Davis IRB policies. Subjects were excluded if injury occurred > 48 h prior to admission or if wound debridement occurred prior to the initial sample collection. Subjects remained on the study for the first 28 days of admission to the BICU or until discharge from the BICU, if occurring prior to 28 days. The course of clinical treatment was not altered for any subject on the study.

#### Healthy volunteers

A cohort of 18 healthy adult (> 18 years old) volunteers from UCDH were consented, in accordance with UC IRB policies, for a single timepoint skin swab collection on the right anterior forearm. This cohort served as a method validation for sequencing from premoistened swabs on healthy skin, and as a comparison to the burned cohort. The health status of volunteers included in this cohort was determined by the following criteria: no hospitalization in the last 30 days or antibiotic use in the last 14 days.

#### Burn nurses

A cohort of five BICU nurses who actively participated in routine patient care were consented and enrolled for a single timepoint skin swab collection on the right anterior forearm. Volunteers were included if they were an active BICU nurse with no recent (< 14 days) history of antibiotic use.

### Sample and data collection

#### Microbial sampling

Bacterial populations were sampled using a sterile rayon swab (BD BBL Culture Swab, Media-free, BD, Franklin Lanes, NJ, USA) premoistened in sterile saline (Research Products International Corp, Mount Prospect, IL, USA). Burn subjects were sampled at the following four locations: spared skin, burn wound, perianal, rectal. Skin sampling was performed by spinning the swab for 30 s across a 4 cm^2^ area while applying moderate pressure^11^. Following collections, the swab tip was placed in a sterile 1.5 ml microcentrifuge tube (Eppendorf Snap-Cap Microcentrifuge Biopur Safe-Lock, Eppendorf, Hamburg, Germany) and stored at -70 °C. Working surfaces were sterilized with 70% ethanol between each sample. Burn wound samples were collected during wound care following removal of dressings and topical treatments but prior to disinfection with dilute chlorohexidine gluconate (4.0% w/v). Perianal and rectal samples were collected by nurses according to UCDH protocol. The “burn wound” and “spared skin” sites were determined for each subject based on the following criteria (Figure S1):

- Partial or full thickness burn present at the burn site
- Healthy and burned skin sites were bilaterally symmetrical from each other across the sagittal plane of the body
- Healthy skin was a minimum of 10 cm from burned tissue
- Neither site was expected to be obstructed by line placement or clinical equipment
- The healthy site was not expected to be utilized as a donor site for autologous skin grafting

#### Longitudinal design

**Figure 1:**
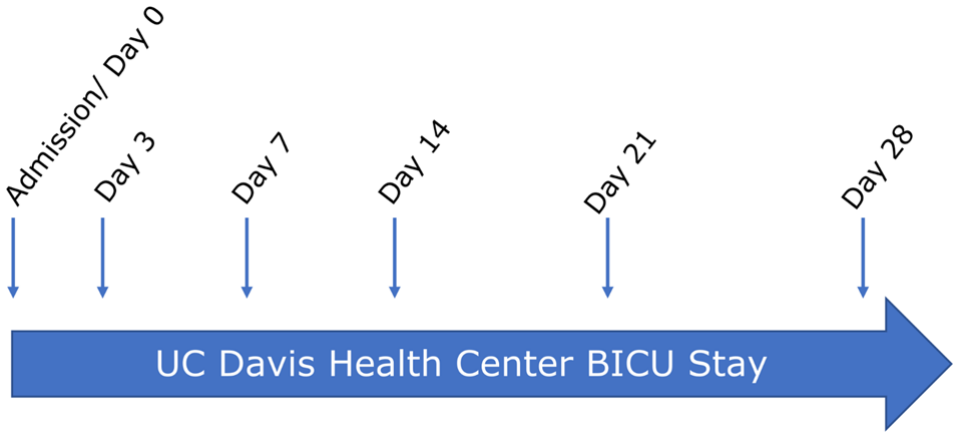
Longitudinal Sampling Schedule. This figure was generated by the author using the software Inkscape^12^.

One swab from each of the four sites was collected on approximately day 0, 3, 7, 14, 21, and 28 post admission of the subject’s stay in the UCDH BICU (Fig. 1). Swabs were collected until day 28 or up to discharge, if prior to 28 day, with 48-h variance from the target timepoint to accommodate clinical workflow.

#### Patient demographics

The following metadata patient demographics were recorded for each subject throughout BICU admission: age, gender, percent TBSA, burn depth, mechanism of injury, time of injury, intubation status, topical and systemic antibiotic dosing, dressings applied during wound care, microbiology cultures, infection, sepsis, nutrition, graft adhesion or failure, wound care frequency and type, and mortality.

### Library generation and sequencing

#### DNA isolation

DNA was isolated from swabs using the DNeasy PowerSoil Kit (Qiagen, Valencia, CA, USA) with modifications. Samples were pre-processed by adding solution C1 to the PowerBead tubes and vortexing. Three hundred microliters of this solution were pipetted into the sample collection tube containing the swab tip and incubated at 37 °C for one hour. The collection tube was then centrifuged with a spin basket (Spin-X centrifuge tube filters, Corning Costar Corporation, Cambridge, MA, USA), and the flow through pipetted back into the PowerBead tube. Further steps followed the manufacturer’s protocol with the exception of adjusting the final elution volume to 50 µl. All modifications were performed to maximize DNA yield as cutaneous sampling yield low microbial biomass^13^. Extracted DNA was stored at − 20 °C.

#### Control samples

One control mock specimen, designated “reagent control”, was processed in parallel with each set of subject’s samples. Reagent controls were prepared at the laboratory immediately prior to DNA isolation by aseptically clipping a sterile swab into a collection tube and proceeding with extraction. Additionally, three “air control” samples were obtained during the wound care of burn subjects on day 3 prior to study sample collection. Air control swabs were spun in the air for 30 s to sample potentially aerosolized bacteria post dressing removal.

#### 16S amplicon library generation

16S metagenomic sequencing libraries were prepared at the Genomics Shared Resource (Sacramento, CA, USA) according to Illumina’s (Illumina, San Diego, CA, USA) 16S Metagenomic Sequencing Library Preparation protocol (Part# 15044223 Rev. B). A universal primer set was used to amplify the V3–V4 hypervariable region^14^, generating a 550 base-pair amplicon: Forward Primer = 5′ TCGTCGGCAGCGTCAGATGTGTATAAGAGACAGCCTACGGGNGGCWGCAG and Reverse Primer = 5′ GTCTCGTGGGCTCGGAGATGTGTATAAGAGACAGGACTACHVGGGTATCTAATCC^11^. Primer pairs obtained from the Access Array Barcode Library for Illumina Sequencers were hybridized to the amplicons by a second round of PCR. Nucleic acid yields were measured by fluorometric quantification using the Qubit 2.0 with the Qubit dsDNA HS Assay Kit (Life Technologies, Carlsbad, CA, USA).

#### Sequencing

Libraries were pooled to a concentration of 118 ng/uL and volume of 40 uL for sequencing on the Illumina MiSeq platform (Illumina, San Diego, CA, USA). These libraries were sequenced using MiSeq v3 chemistry with 300-bp paired end reads at the UC Davis DNA Technologies and Expression Analysis Core (Davis, CA, USA).

### Data analysis

#### Bioinformatics

Bacterial 16S rRNA gene sequence data were analyzed with the open source software Quantitative Insights Into Microbial Ecology 2 (QIIME 2) version 2020.2^15^. Raw sequence data were demultiplexed (via q2-demux) and paired end reads were quality filtered, trimmed to a minimum median quality score of 20, and chimeric sequences removed using the Divisive Amplicon Denoising Algorithm 2 (DADA2)^16^. Sequence alignment was performed using Multiple Sequence Alignment Software Version 7 (MAFFT)^17^ (via q2‐alignment) and a rooted phylogeny generated with FastTree 2^18^ (via q2‐phylogeny).

Diversity analysis was performed using q2‐diversity following rarefication by subsampling without replacement to 1156 sequences per sample. Alpha and beta diversity metrics were calculated by Shannon Diversity and Bray‐Curtis dissimilarity, respectively^19,20^. Taxonomy was assigned using the q2‐feature‐classifier^21^ classify‐sklearn Naïve Bayes taxonomy classifier against the Greengenes 13_8 99% Operational Taxonomic Units full length reference sequences^22^. This classifier was trained to the hypervariable V3–V4 region using the primers previously described. Differential abundance was determined by Analysis of Composition of Microbiomes (ANCOM) and q2-ALDEx2^23,24^. Two methods for determining enriched or depleted taxa are reported due to the current lack of consensus on which multivariate analytical practice is most appropriate for microbial data. The QIIME2 Longitudinal (q2-longtitudinal) plugin compared site-specific subject sampling over time for metrics of alpha diversity, beta diversity, and taxonomy^25^.

#### Statistical analysis

Non-parametric Kruskal–Wallis test performed in QIIME 2 determined alpha diversity significance (via q2-diversity) between subject groups, sampling location, and metadata parameters^26^. Principal coordinate analysis (PCoA) plots were generated as visualizers for beta diversity measures and statistical analysis performed with Permutational Multivariate Analysis of Variance (PERMANOVA)^27^ test for between metric significance. Alpha and beta diversity visualizations were generated using QIIME 2, qiime2R, and R Studio^28,29^. Differential taxonomic abundance determined by ALDEx2 was considered significant with a false discovery rate adjusted *P* value of < 0.05.

#### Contaminant identification and removal

Contaminants can arise during collection, nucleic acid isolation, PCR amplification, and during sequencing by lane cross contamination^30,31^. We employed the recommendations established by Salter et al. to reduce and identify contaminants. Any sequence meeting these guidelines was filtered out from analysis (via q2-demux)^15,32^.

## Results

### Subject demographics

The study dataset comprised of 10 male burn patients admitted to the UCDH BICU, 18 healthy volunteers working at UCDH but not in the BICU, and 5 female BICU nurses. Burn patients were aged 31–61 years (median 38 years) and suffered a median TBSA burn of 26.5% (range 10–76%). Of the 10 burn patients, 50% (*n* = 5) required grafting of the wound sampling location, 50% (*n* = 5) experienced bacterial and/or fungal wound infections, 20% (*n* = 2) were diagnosed with culture positive bacterial pneumonia, and 20% (*n* = 2) with blood culture positive septicemia. There were no instances of diagnosed inhalation injury (Table 1). Median time from BICU admission to the first sample collection was 5 h (range 1–14 h) and the median number of days on study was 21 (range 7–28 days). The healthy volunteer cohort consisted of 10 female and 8 male volunteers and the burn nurse cohort was 100% female (*n* = 5).

**Table 1:** Burn patient demographics.

Subject ID	Age (years)	Total TBSA burn (%)	Race or ethnicity	Mechanism of injury	Time on study (Days)	Burn depth at collection location	Burn site grafted (y/n)	Culture positive (y/n)	Day(s) of culture(s)	Organism(s) cultured (location)
S1	37	36	Hispanic	Flame	21	Partial-thickness	n	n	NA	NA
S2	34	16	White	Flame	7	Full-thickness	y	n	NA	NA
S3	34	22	White	Flame	14	Partial-thickness	n	y	3	MRSA (B/W)
S4	61	18.5	Native American	Flame	21	Partial-thickness	n	y	9	*Klebsiella *sp. (R)
S5	39	20	White	Flame	14	Full-thickness	y	y	8	MRSA (W)
S6	31	40	Other	Flame	28	Partial-thickness	n	y	3	Enterobacter cloacae complex (W)
									12	*Lactobacillus *sp. (W)
									14	*Streptococcus pneumonia*, *Stenotrophomonas maltophilia* (R)
S7	36	76	Hispanic	Scald	28	Partial-thickness	n	y	6	*Lactobacillus *sp.*;* yeast (W)
									9	*Candida albicans* (B)
S8	43	31	Hispanic	Flame	28	Full-thickness	y	n	NA	NA
S9	58	40.5	White	Flame	14	Full-thickness	y	n	NA	NA
S10	43	10	White	Flame	28	Full-thickness	y	y	7	*Staphylococcus aureus* (W)

*B* blood, *R* respiratory, *W* wound.

### Sampling and bioinformatic filtering

Of the total collected samples (*n* = 228), 210 were included in the dataset. Twelve samples were excluded pre-sequencing due to undetectable nucleic acid concentrations following extraction and six samples were excluded bioinformatically with < 20% of sequences passing quality filters and/or achieving a sampling depth of < 1156 (Table 2). Following recommendations from Salter et al. one potential contaminant amplicon sequence variant (ASV), a unique DNA variant in the dataset, mapping to *Ralstonia* species (sp.) was identified^33^. This genus has been reported as a reagent contaminant and appeared in all low abundance samples following implementation of a new extraction kit (Figure S3). The ASV was removed from the dataset by filtering with q2-demux—all results reported are in exclusion of this taxon.

**Table 2:** Total samples included in analysis.

Collection group	Samples collected	Samples post extraction	Total included	%Excluded
Burn	48	46	44	8.3
Spared	49	44	44	10.2
Perianal	47	46	45	4.4
Rectal	48	48	46	4.2
Reagent control	10	6	5	50
Air control	3	3	3	0
Nurse	5	5	5	0
Healthy volunteer	18	18	18	0
Total	228	216	210	7.9

### Sample group diversity and taxonomy

#### Taxonomic relative abundance

**Figure 2:**
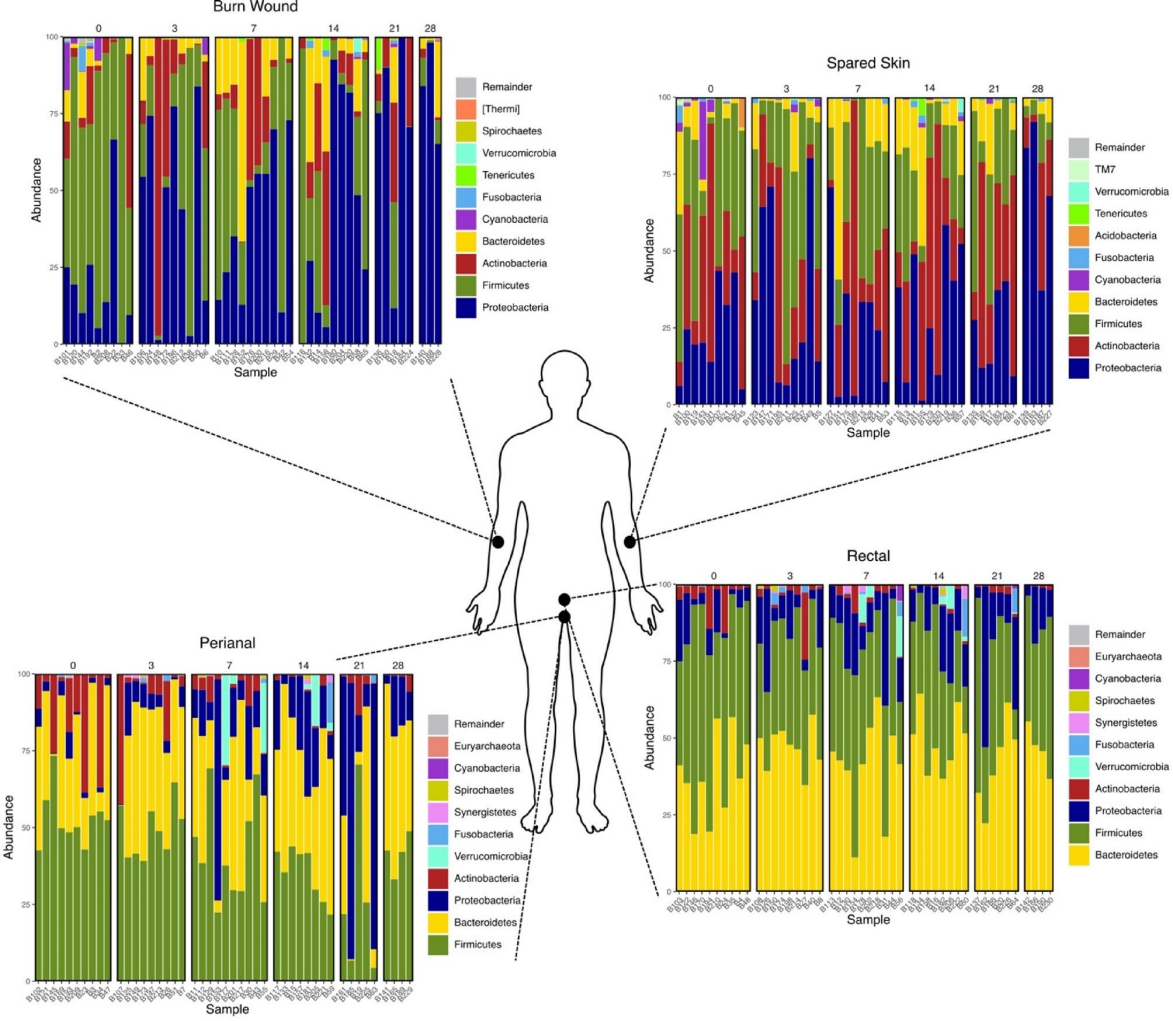
Burn patient taxonomic summary. Summary of taxonomic relative abundance consisting of the top 10 phyla sequenced from samples collected at four collection locations (burn wound, spared skin, perianal, and rectal) on the burn patient cohort displayed by collection day. The numbers at the top of each panel represent the collection timepoint (“0” represents samples collected on day 0, etc.). Phyla are listed descending from least to most abundant. This figure was generated using the software Inkscape and graphical taxonomic output from qiime2R.

Sample group taxonomy was collapsed by collection location across all burn subjects and sampling timepoints with phylum level classification (Fig. 2). The four most prevalent genera sampled from the burn patient wound (*Staphylococcus*, *Burkholderia*, *Stenotrophomonas*, and *Propionibacterium*) were also predominant on burn patient spared skin in varying proportions (Table 3). Analysis of differential feature abundance of features (q2-ALDEx2) found four ASVs with an effect size > 1 between burn wound and spared skin (Figure S4). Of the four features identified by ALDEx2, three mapped by the National Center for Biotechnology Information (NCBI) Basic Local Alignment Search Tool (BLAST) with 100% identity to strains of *Propionibacterium acnes* and one to *Staphylococcus epidermitis*, both are reduced on the burn wound. Perianal and rectal sampling locations did not exhibit significantly different taxa when compared by ANCOM and ALDEx2.

**Table 3:** Predominant taxa by sample group.

Subject	Sample group	Phyla (% abundance)	Genera (% abundance)
Burn patient	Wound	Proteobacteria (56.9)	*Staphylococcus* (23.7)
		Firmicutes (27.2)	*Propionibacterium* (8.3)
		Actinobacteria (8.0)	*Stenotrophomonas* (7.3)
		Bacteroidetes (6.1)	*Burkholderia* (3.1)
	Spared skin	Proteobacteria (43.0)	*Propionibacterium* (25.7)
		Actinobacteria (24.9)	*Staphylococcus* (12.2)
		Firmicutes (22.4)	*Stenotrophomonas* (3.0)
		Bacteroidetes (7.6)	*Burkholderia* (2.9)
	Rectal	Bacteroidetes (42.6)	*Prevotella* (12.9)
		Firmicutes (39.4)	*Bacteroides* (11.6)
		Proteobacteria (13.0)	*Porphyromonas* (7.6)
		Actinobacteria (2.8)	*Campylobacter* (6.0)
	Perianal	Firmicutes (42.7)	*Bacteroides* (10.1)
		Bacteroidetes (32.8)	*Prevotella* (7.9)
		Proteobacteria (15.6)	*Porphyromonas* (6.8)
		Actinobacteria (6.2)	*Campylobacter* (2.8)
Healthy volunteer	Healthy skin	Actinobacteria (48.2)	*Propionibacterium* (18.1)
		Proteobacteria (23.4)	*Corynebacterium* (16.1)
		Firmicutes (21.6)	*Staphylococcus* (6.9)
		Bacteroidetes (3.4)	*Burkholderia* (6.1)
BICU nurse	Healthy skin	Proteobacteria (50.8)	*Stenotrophomonas* (29.6)
		Actinobacteria (35.1)	*Propionibacterium* (15.6)
		Firmicutes (10.8)	*Corynebacterium* (8.7)
		Bacteroidetes (2.1)	*Burkholderia* (8.0)

In descending order, the top four most predominant phyla and genera as determined by percent relative abundance summed by sample group among burn patients, healthy volunteers, and BICU nurses. Percent abundance represents the frequency of each phyla or genera divided by the sum of frequencies for each group.

**Figure 3:**
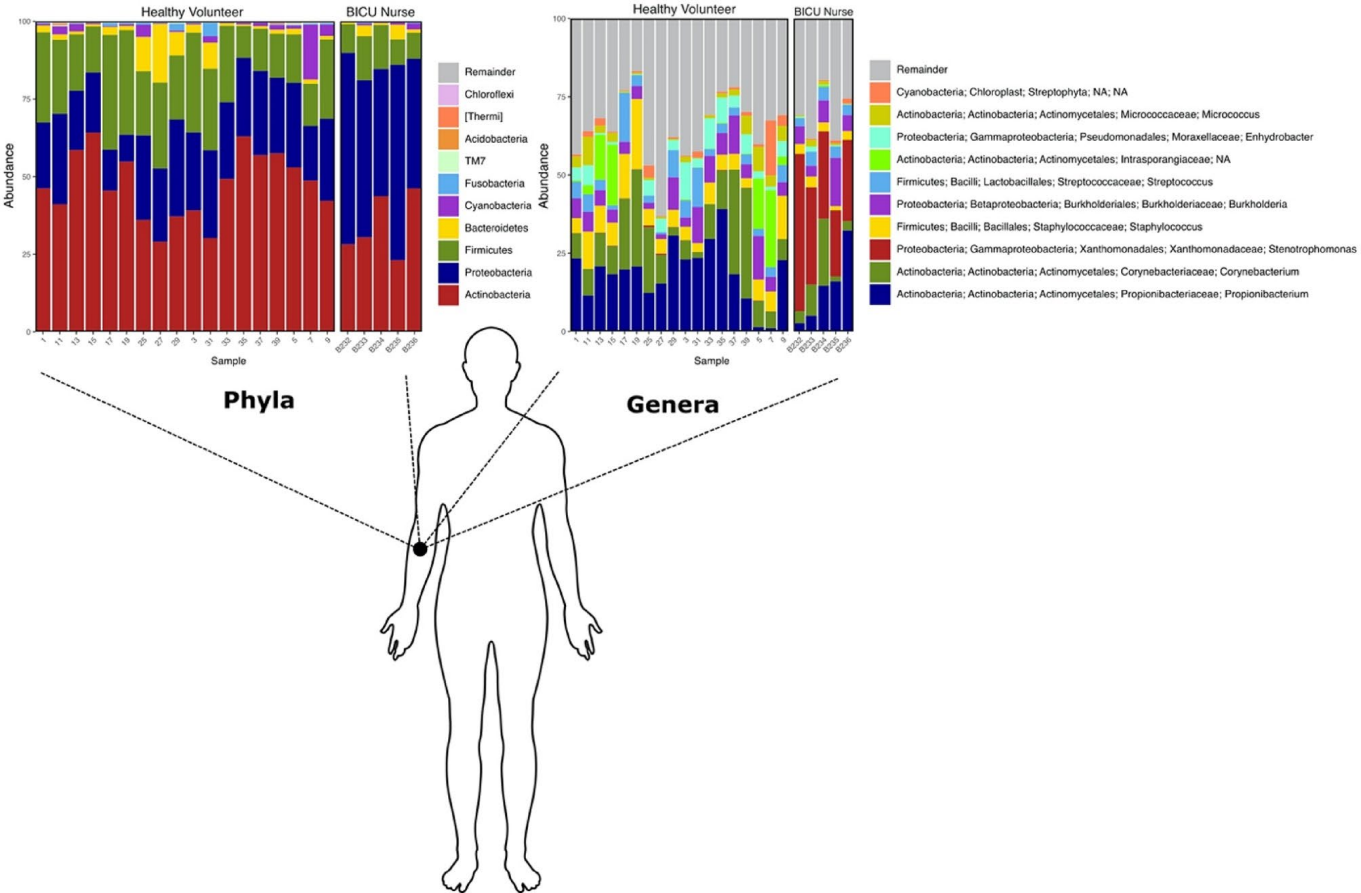
Healthy volunteer and BICU nurse taxonomic summary. Summary of the relative abundance of the top 10 phyla and genera level taxa on the healthy volunteer and BICU nurse cohorts. Phyla and genera are listed descending from least to most abundant. This figure was generated using the software Inkscape and graphical taxonomic output from qiime2R.

Similarly, sample group taxonomy was collapsed by healthy volunteer and BICU nurse cohorts by phylum and genus level classification (Fig. 3). Eleven ASVs exhibit differential relative abundances between BICU nurses and healthy volunteers. These ASVs mapped by NCBI BLAST to the following taxa by ≥ 99.78% identity: *Stenotrophomonas maltophilia*, *Escherichia coli*, *Stenotrophomonas *sp., *Variovax paradoxus*, *Sphingomonas *sp., *Staphylococcus epidermidis*, *Pseudomonas *sp., and *Microbacterium *sp. With the exception of *Staphyloccus epidermidis*, all are enriched on BICU nurse samples.

**Figure 4:**
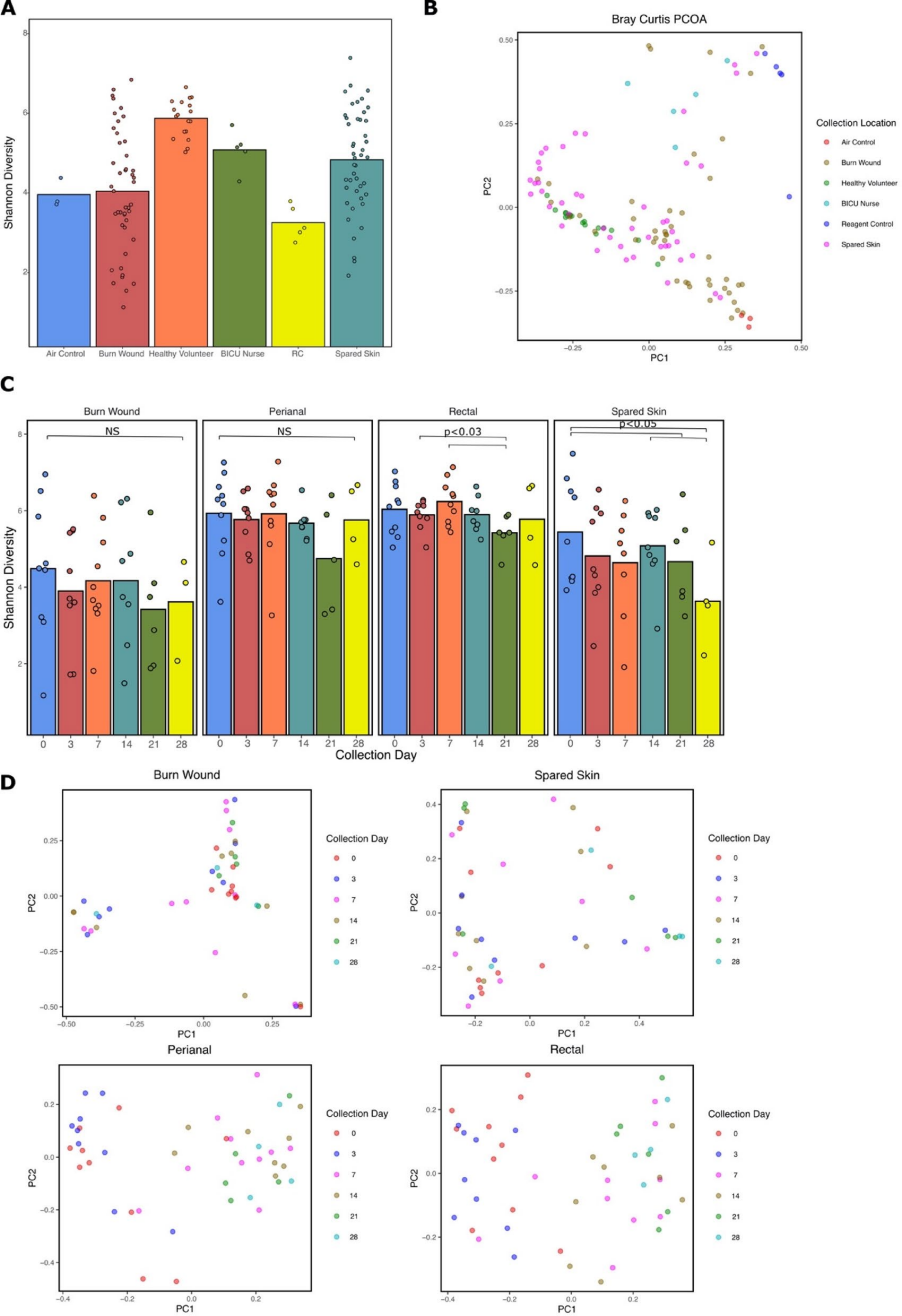
Diversity and Taxonomy of Skin and Gut Microbiota. (**A**) Mean Shannon Diversity Index from cutaneous sampling locations and control swabs. (**B**) Compositional community analysis by Bray Curtis dissimilarity demonstrate clustering by sample type (*P* = 0.001) between skin and control samples. The first and second principal components (PC1 and PC2) are shown, explaining 13.94% and 11.25% of the variance in the dataset, respectively. Each color-coded point on the graph correlated with a subject body site or control type. (**C**) Shannon Diversity displayed over sampling days 0, 3, 7, 14, 21, and 28 for samples collected at the burn wound, spared skin, perianal, and rectal sampling sites of the burn patient cohort. Burn wound and perianal Shannon diversity does not significantly differ between collection days (*P* = 0.868 and *P* = 0.046, respectively). Rectal sample diversity does not significantly differ by collection day (*P* = 0.222); however, pairwise comparisons between days 3 and 21 (*P* = 0.025) and days 7 and 21 (*P* = 0.013) are significantly different. Spared skin diversity by collection day is not significant by Kruskal Wallis between all groups (*P* = 0.089); however, pairwise tests are significantly different between days 0 and 21 (*P* = 0.251), days 0 and 28 (*P* = 0.031), and days 14 and 28 (*P* = 0.021). (**D**) Compositional community analysis by Bray Curtis dissimilarity by collection day for each sampling location. In the burn wound plot, PC1 and PC2 explain 14.8% and 13.31% of the variance, respectively. In the spared skin plot, PC1 and PC2 explain 21.45% and 14.18% of the variance, respectively. Burn wound and spared skin samples did not exhibit significant clustering by collection day (*P* = 0.106 and *P* = 0.403, respectively) by PERMANOVA. In the perianal plot, PC1 and PC2 explain 15.6% and 7.062% of the variance, respectively. In the rectal plot, PC1 and PC2 explain 17.08% and 7.367% of the variance, respectively. Rectal and perianal sites exhibit significant clustering (*P* = 0.001 PERMANOVA). Pairwise PERMANOVA testing between days 0 and 3 are not significant to each other (*P* > 0.05) but are significantly different from days 7, 21, and 28 (*P* < 0.03). Similarly, days 7, 21, and 28 are not significantly different from each other (*P* > 0.05 pairwise PERMANOVA) but significantly different from days 0 and 3 (*P* < 0.03 pairwise PERMANOVA). *RC* reagent control, *NS* not significant. This figure was generated using the software Inkscape and visualizations generated by qiime2R.

Between-sample diversity, as measured by Shannon Diversity, is significantly different between all group comparisons (*P* < 0.05) except between air controls/burn wound (*P* = 0.487), air controls/nurses (*P* = 0.052), and burn wound/nurses (*P* = 0.325) (Fig. 4A). Healthy volunteers have significantly higher mean Shannon diversity compared to cutaneous burn samples and nurses (*P* < 0.01). Bray Curtis dissimilarity distance of microbial abundance differed significantly between collection groups (*P* = 0.001 PERMANOVA) (Fig. 4B). Shannon diversity is not significantly different between the days of collection for samples collected from the burn wound and perianal sites. Rectal samples collected on day 21 have reduced Shannon diversity compared to days 3 and 7 (*P* = 0.025 and *P* = 0.013, respectively). The Shannon diversity from spared skin appears to reduce over time (Fig. 4C). When beta diversity is explored longitudinally, the gut samples collected from days 0 and 3 are significantly different from those collected on days 7, 14, 21, and 28 when compared by pairwise PERMANOVA (Fig. 4D).

### Effects of burn treatments on diversity

#### Antimicrobials

**Figure 5:**
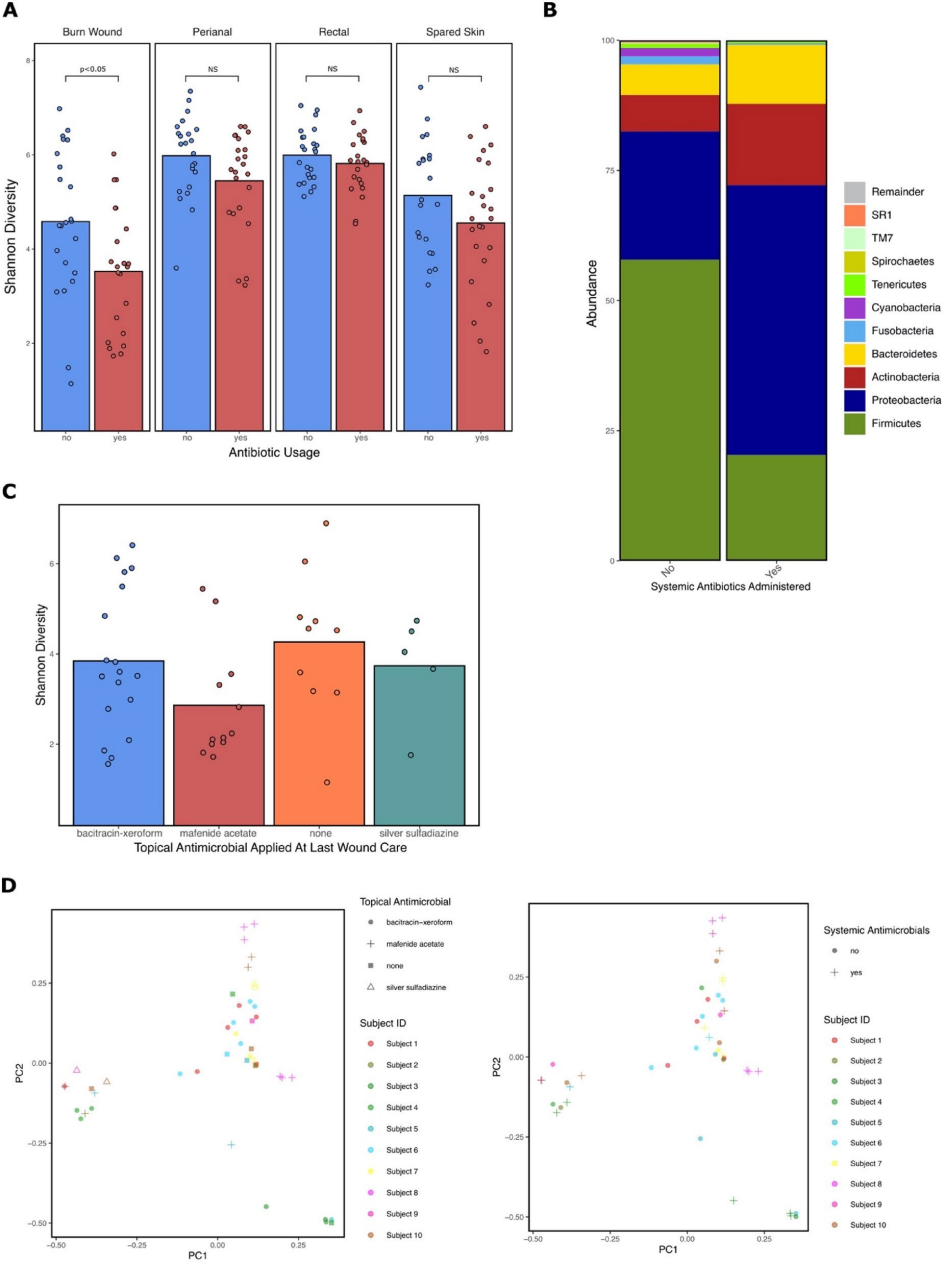
Impact of systemic and topical antibiotics on microbial diversity and taxonomy. (**A**) Mean Shannon Diversity of samples collected prior to systemic antibiotic administration and post for each collection location across the burn patient cohort. Microbial diversity at the burn wound is significantly reduced (*P* < 0.05) following systemic antibiotic administration. The spared skin, rectal, and perianal collection locations were not significantly altered. (**B**) Taxonomic summary of the top 10 phyla sequenced from burn wound samples collected pre- and post-systemic antibiotic administration. SR1 and TM7 indicate candidate phyla that have been identified by sequencing but have no cultivated examples. (**C**) Mean Shannon Diversity of burn wound samples collected after clinical application of differential topical antimicrobials. (**D**) Bray Curtis PCOA plots for topical and systemic antimicrobial administration on the burn wound. The first and second principal components (PC1 and PC2) are shown, explaining 14.8% and 13.31% of the variance, respectively. Type of topical antimicrobial applied at the last wound care results in significant between sample diversity between mafenide acetate and silver sulfadiazine (*P* = 0.007). Clustering by systemic antibiotics administered prior to collection was not significant (*P* = 0.093). *NS*, not significant. This figure was generated using the software Inkscape and visualizations generated by qiime2R.

Systemic antimicrobials were administered to 100% of the burn study population at one or more timepoints. Samples collected following systemic antibiotic administration were clustered by collection location and compared to samples collected prior to any systemic antibiotic administration. The burn wound exhibits significantly reduced alpha diversity (*P* = 0.023) following systemic antibiotic administration (Fig. 5A). Analysis with ANCOM and ALDEx2 did not reveal any significantly different taxa on the burn wound samples following systemic antibiotic administration. However, the relative abundance of the phylum Firmicutes decreased (57.9–20.3%) following antibiotic administration while Proteobacteria increased (24.6–51.8%) (Fig. 5B). Application of different topical antimicrobials during the wound care prior to sample collection did not differentially impact alpha diversity of the burn wound populations (Fig. 5C) and were not significantly different from samples collected on admission, prior to the first wound care where topicals were initially applied. Between sample diversity on the burn wound, analyzed by Bray Curtis dissimilarity, was not significantly different between subject’s that received systemic antimicrobials and those that did not prior to collection. The type of topical antimicrobial applied did impact beta diversity between mafenide acetate and silver sulfadiazine (*P* = 0.007) (Fig. 5D).

#### Grafting

**Figure 6:**
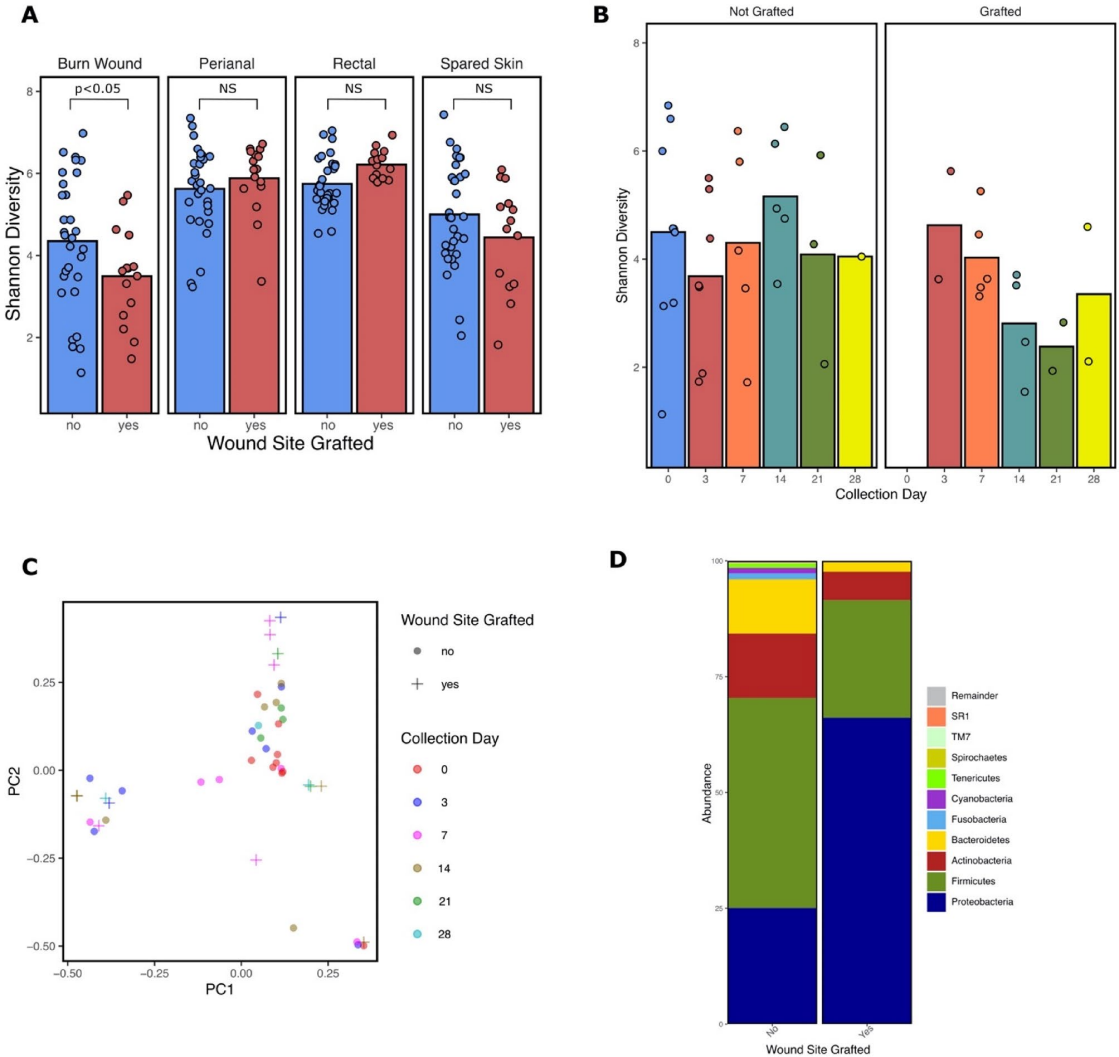
Effects of grafting on burn wound alpha diversity and taxonomy. (**A**) Mean Shannon Diversity from each sampling location (burn wound, spared skin, perianal, and rectal) clustered by presence or absence of grafting at the wound sampling location. Diversity at the burn wound is significantly reduced when grafted. (**B**) Mean Shannon Diversity from the burn wound samples over time clustered by wound grafting status. (**C**) PCOA plot of Bray Curtis dissimilarity of the burn wound samples displaying grafting status and collection day. The first and second principal components (PC1 and PC2) are shown, explaining 14.8% and 13.31% of the variance, respectively. Samples cluster significantly by PERMANOVA by grafting status (*P* = 0.002). (**D**) Taxonomic summary of the top 10 phyla sequenced at the burn wound and clustered by wound grafting status. *NS*, not significant. This figure was generated using the software Inkscape and visualizations generated by qiime2R.

Autologous skin grafting occurred at the sampling site in 50% of the burn study population (*n* = 5) affecting 33.3% of all burn wound samples (*n* = 15). Samples obtained from grafted skin exhibited reduced alpha diversity compared to non-grafted samples (*P* = 0.036) while grafting had no significant effect on the alpha diversity at other collection locations (Fig. 6A). When visualized longitudinally, alpha diversity trends towards recovery by day 28 in grafted samples (Fig. 6B). Beta diversity differs by grafted and non-grafted burn wound sites (*P* = 0.002), however, does not significantly cluster by the day of collection when displayed by PCOA (Fig. 6C). Analysis by ALDEx2 did not classify any ASVs as significantly different between grafted and non-grafted wound samples. Analysis by ANCOM revealed the phylum Fusobacteria to be significantly reduced among the grafted wound samples (W = 14). The relative abundance of Proteobacteria increased from 25.1 to 66.2% following grafting while Firmicutes decreased from 45.3 to 25.4% (Fig. 6D). Taxonomic differences between samples collected at day 0 for each swab location were investigated to determine if any taxa are significantly different on admission for those who later required grafting. Analysis by ANCOM found the phylum Verrucomicrobia to be enriched on the spared skin of grafted subjects (W = 35) as well as enrichment of the genera *Streptococcus* (W = 21) and *Actinomyces* (W = 4) from the perianal swabs of grafted individuals. No significantly different taxa were identified from the burn wound or rectal collection sites on day 0 between the two groups.

#### Diet

**Figure 7:**
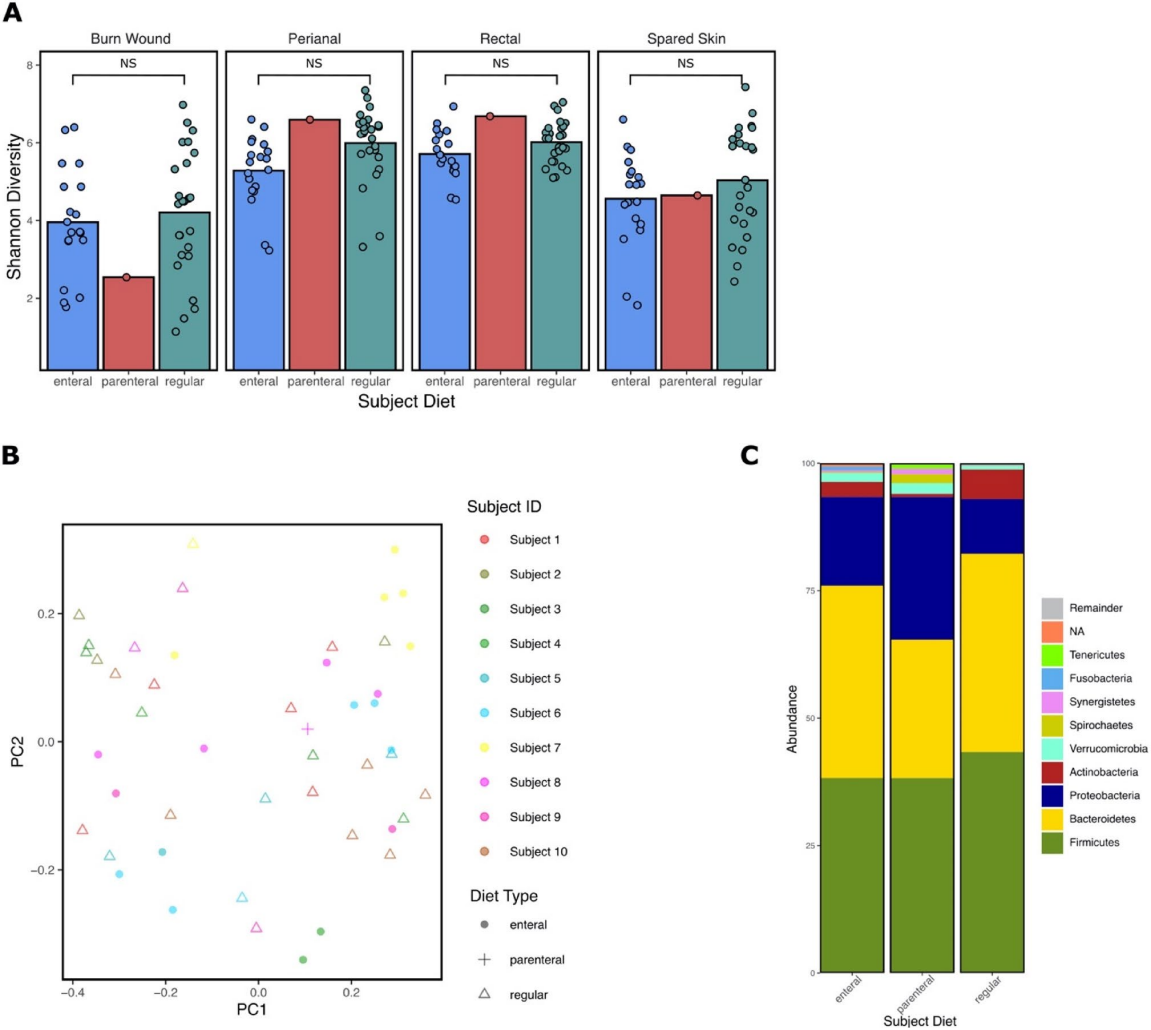
Nutritional impacts on burn patient microbial diversity and taxonomy. (**A**) Mean Shannon Diversity between diet type that each patient received during the collection timepoint for each collection location (burn wound, spared skin, perianal, and rectal) sampled. (**B**) PCOA plot of Bray Curtis dissimilarly of rectal samples illustrates compositional diversity complicated by significant within group variation (*P* = 0.036) and inter-subject variation (*P* = 0.001). The first and second principal components (PC1 and PC2) are shown, explaining 17.08% and 7.367% of the variance, respectively. (**C**) Taxonomic summary of the top 10 phyla sequenced from the sum of perianal and rectal samples separated by diet type at the time of collection. “NA” represents unclassified phyla. This figure was generated using the software Inkscape and visualizations generated by qiime2R.

The effects of diet type (enteral, parenteral, or regular) were minimal on alpha diversity between collection locations (Fig. 7A). Bray Curtis dissimilarity between diet types indicate that gut samples derived from patients receiving enteral nutrition are not significantly compositionally dissimilar to those collected from patients consuming a regular diet (Fig. 7B). Analysis of differential relative abundance by ANCOM found the genera *Proteus*, *Enterococcus*, and *Methanobrevibacter* to be significantly enriched in patients receiving enteral nutrition (W = 180, W = 174, W = 174, respectively) compared to a regular diet (Fig. 7C and Figure S4). ALDEx2 did not identify significantly different ASVs between the groups.

### Detection of clinically identified infections

**Figure 8:**
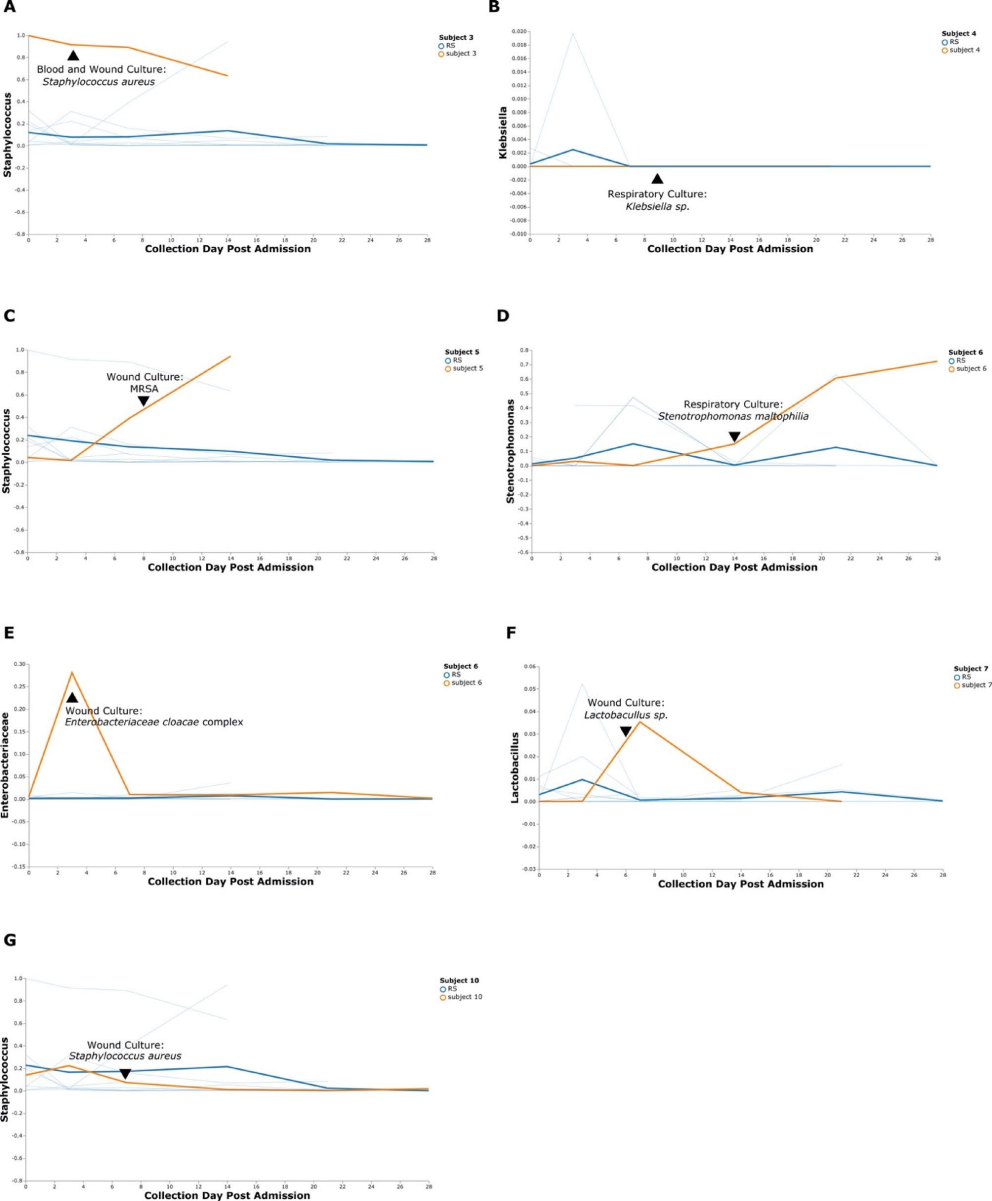
Longitudinal Visualization of Culture Isolated Bacteria from the Burn Wound. (**A**–**G**) Volatility plots display in orange the longitudinal change relative abundance of a bacterial ASV from the order or genus that was identified by culture for individual burn subjects. The thick blue line depicts the average of the relative abundance of the same ASV from the remaining subjects. Thin blue lines are representative of individual relative abundances of the ASV from each of the remaining subjects. A black triangle indicates the day a culture was collected for a clinically suspected infection for each subject while on the study. Subjects 3, 4, 5, 7, and 10 had one culture and Subject 6 had two. *MRSA*, methicillin resistant *Staphylococcus aureus*; *RS*, remaining subjects. This figure was generated using the software Inkscape and visualizations generated by QIIME2.

Six of 10 burn patients experienced culture positive infection at one or more timepoints during the study. Nine instances of bacterial infection were detected in wound, blood, and respiratory cultures occurring across these six patients. Burn wound samples were evaluated for the family or genus of the infecting organism and plotted by relative abundance to detect changes over time. Irrespective of culture location, 55.6% (5/9) of infections were detectable by an increase in relative abundance on the burn wound from the single sample site collected (Fig. 8). Notably, for Subject 6 (Fig. 8D), *Stenotrophomonas maltophilia* was cultured from a respiratory sample on day 14 and clinically treated for pneumonia. On this subject’s wound, the genera *Stenotrophomonas* was undetectable on admission, however on day 14 consisted 15.1% of ASVs sequenced. *Stenotrophomonas* remained a dominant taxon on the wound comprising 60.6% and 72.3% of ASVs on days 21 and 28, respectively, despite systemic antibiotic administration for pneumonia on day 14. Diversity metrics and taxonomy were evaluated at samples collected on admission to determine if any features could be identified for subjects that later develop infection. No significant differences in diversity were identified between these groups (Figure S7).

## Discussion

Previous studies have profiled the human skin microbiome reporting differential populations and diversity found on the human skin; however the majority still support one of the sentinel molecular 16S sequencing studies that found that the human forearm consists predominantly (94.6% of clones) of the phyla *Actinobacteria*, *Firmicutes*, and *Proteobacteria*^34^. These data are supported by our findings for the control populations of healthy volunteers and BICU nurses sampled on the right forearm as well as the burn patient wound and spared skin samples (Table 3). Despite partial and full-thickness tissue destruction, the burn wound samples still have predominant phyla reflective of an uninjured skin microbiome, which demonstrates appropriate sampling for skin bacteria.

When investigating diversity metrics, we found that burn wound microbial populations were significantly less diverse compared to spared skin, healthy volunteers, and BICU nurses (Fig. 4A). Reduction in microbial population alpha diversity has been reported as a characteristic of non-healing wounds and could have implications for healing rates in the burn population^35^. We expected to see an initial reduction and subsequent recovery of alpha diversity from the wound sites, however this was not supported (Fig. 4C). While these findings were unexpected, a study investigating in chronic wound healing of diabetic foot ulcers suggests that temporal stability in wound microbiome are indicative of poorly healing chronic wounds^36^. The sustained reduction in alpha diversity could be due to frequent routine application of topical antimicrobials or differential healing in our population, among other factors not controlled for in this study. *Propionibacterium acnes* and *Staphylococcus epidermitis* are two skin commensal organisms that were significantly reduced on the wound site, supporting that burn wounds experience perturbance of normal commensal bacteria. Rectal and perianal sample alpha diversity did not significantly change over time. This contradicts findings by Wang et al. who demonstrated initial reduction in alpha diversity following severe burn injury and progressive recovery over six weeks of sampling from seven subjects^37^. Evaluation of between sample diversity for each sampling location found that gut representative samples, rectal and perianal, change over time with timepoints forming two clusters across all subjects: those collected from days 0 and 3 and those collected from days 7, 14, 21, and 28 (Fig. 4D). These findings suggest that the gut microbial profile did change over time however, within sample species numbers and evenness was not affected in this population.

Interestingly, the diversity and composition of the BICU nurse microbiome was significantly different compared to healthy volunteers not working in the BICU. Relative enrichment of *Stenotrophomonas *sp. and reduction in the normal commensal *Staphylococcus epidermitis* suggest that nurses experience a change in community composition of their cutaneous microbiota. Isolation protocols such as frequent sanitization of the hands and forearms could impact the bacterial populations. In addition, the patient population nurses interact with could influence the cutaneous microbiota, leading to disparities between nurses working in different units. Health care worker hand microbiota has been suggested to mediate the transfer of pathogenic bacteria with frequent hand washing being associated with potential pathogen carriage^38^. Loftus et al. found hand contamination by anesthesia workers to be a source of intraoperative bacterial transmission to the intraoperative environment^39^. Further research is warranted in this area as our study has a small sample size of five nurses with a female gender bias.

Our findings suggest that systemic antimicrobial administration significantly reduces microbial diversity at the burn wound but does not impact other microbial populations evaluated in the study (Fig. 5A). Woo et al. found cutaneous microbial alpha diversity to be unaffected in Rosacea patients administered systemic antibiotics^40^. The relative decrease in Firmicutes and increase in Proteobacteria following administration could be a result of broad-spectrum coverage against Gram-positive organisms allowing for Gram-negative enrichment. The type of topical antimicrobial applied on the burn wound did not significantly impact alpha diversity or taxonomic composition at the wound, but beta diversity differed between wounds receiving mafenide acetate and silver sulfadiazine (Fig. 5D). These findings are supported by SanMiguel et al. who report that topical antibiotics reduce colonization of *Staphylococcus aureus* but only triple antibiotic ointment reduces metrics of microbial diversity on the skin^41^. Grafting of a burn wound and the clinical process associated with the procedure, such as excision, anesthesia, and prophylactic antibiotics are all proposed to cause community composition changes to microbial populations. We found that diversity is reduced at the wound site following grafting but may trend towards recovery by day 28 (Fig. 6B) as the graft takes and the cutaneous microenvironment becomes more reflective of normal skin. A non-significant decrease in bacterial diversity seen at the spared skin following grafting could be due to the surgery and prophylactic antibiotics associated with the procedure (Fig. 6A). On admission, there was enrichment of certain taxa on subjects requiring grafting from the spared skin and perianal swabs. However, these findings should be further investigated with increased sample size. In addition, the grouping of “grafted” versus “not-grafted” could actually be evaluating burn depth on admission, as deeper burns require grafting.

Adequate nutrition is an essential component of burn care and patients often receive a variety of nutritional sources to meet high caloric needs. We investigated whether the type of nutrition a patient is receiving impacts gut microbial populations and found that enteral nutrition trends towards decreased gut diversity and increased compositional difference compared to samples obtained from patients receiving a regular diet (Fig. 7). These data are not significant and should be further investigated in both burn and non-burn ICU patients. We also found the genera *Proteus*, *Enterococcus*, and *Methanobrevibacter* to be significantly enriched in patients receiving enteral nutrition compared to a regular diet. These findings contradict a study conducted by Wang et al. who found the administration of prolonged enteral nutrition to promote the recovery of gut microbial diversity and the reduction of *Enterococcu*s and *Escherichia*^37^.

The burn population is one that could greatly benefit from early detection of infection with the potential to reduce the incidence of sepsis and subsequent mortality. The ability to monitor common sites of infection, such as the burn wound, for colonization of pathogenic bacteria that could result in local infection or systemic spread would improve targeted antimicrobial therapies and patient outcome. It has been shown that machine learning can be used to predict disease states such as inflammatory bowel disease, type 2 diabetes, and liver cirrhosis from 16S microbial sequencing datasets^42,43^. Additionally, susceptibility to bloodstream infection following chemotherapy has been predicted by the pre-treatment gut microbiome^44^. To investigate this potential use in the burn population we retrospectively tracked changes in relative abundance of organisms over time that were cultured as part of the subjects’ clinical care. We found that local and systemic infections could be seen by the increased abundance of the inciting bacteria at the burn wound (Fig. 8). This patient population should be included in studies evaluating the clinical application of sequencing methodologies and machine learning to detect infection. Comprehensive sampling of multiple sites throughout a patient’s hospitalization could also help indicate the source of infection, which is often unknown in this population.

Studies utilizing 16S microbial community analysis inherently contain limitations. Sample preparation variables such as collection and storage methodology have been shown to influence results^45^. Extraction and amplification of a single section of bacterial DNA can contribute bias to the dataset, as can the sequencing platform used and the bioinformatic algorithms performed during downstream analysis^46^. We aim to acknowledge and address these limitations through the transparency of our methodology and public sharing of the raw sequence data. Specific to this study, a limitation is the burn patient enrollment gender bias towards male participants as well as low sample size due to the pilot nature of the study. Gender was not selected for during enrollment and males are admitted to the ICU for burn injury more frequently than females. In 2006, a ten year review of United States hospital burn admissions found males to be overrepresented, accounting for 67.9%^47^. However, further research should aim for an even gender distribution, as females are at increased risk of death following burn injury and microbial populations have been shown to differ by gender^47,48^. In addition, by nature of 16s rRNA sequencing, functional genomics of the bacterial populations reported in this study could not be determined. In the future, whole genome shotgun sequencing could be employed to determine metabolic capabilities of these microbial populations. Potential future studies should take these factors into account and consider other important aspects of burn care and microbial interactions, such as fluid requirements and healing endpoints.

## Conclusion

This study profiled the microbial populations found on the burn wound, spared skin, perianal, and rectal locations for the first 28 days of 10 burn patients admitted to the UCDH BICU. Our data support the idea that changes in microbial community composition occur at the burn wound and spared skin following injury compared to healthy volunteers, and that BICU nurses may also experience alterations in microbial community composition from cutaneous populations sampled from the forearm. Treatments such as antibiotics, grafting, and nutritional formulations can impact the diversity and community compositions sampled during the patient’s ICU stay. Surprisingly, the topical antimicrobial applied at the wound did not result in a significant difference of relative abundance in any AVSs. In addition, it is possible to detect changes in relative abundance of clinically identified infectious bacteria at the burn wound, even if it is not the site of the culture location. Due to the impact that sepsis can have on patient outcome, further research in establishing burn wound bacterial profiles can aid in the development of tools to detect pathogenic changes.

## Supplementary Information

Supplementary Information.

## Data Availability

The datasets generated during and/or analyzed during the current study are available in the NCBI Sequence Read Archive (SRA) repository, [PRJNA701230], and upon request.
