# Supplementary material for: Longitudinal profiling of the burn patient cutaneous and gastrointestinal microbiota: a pilot study
Source: Sci Rep. 2021 May 21;11:10667. doi: 10.1038/s41598-021-89822-z (PMC8139985; doi:10.1038/s41598-021-89822-z)
Supplement: Supplementary file 1 — Supplementary Information. [file 41598_2021_89822_MOESM1_ESM.docx]

**SCIENTIFIC REPORTS**

**LONGITUDINAL PROFILING OF THE BURN PATIENT CUTANEOUS AND GASTROINTESTINAL MICROBIOTA: A PILOT STUDY**

Kelly M. Lima, BS^1^; Ryan R. Davis, BS^1^; Stephenie Y. Liu, BA^1^; David Greenhalgh, MD, FACS^2^; Nam K. Tran, PhD, HCLD (ABB), FAACC^1*^

^1^Dept. of Pathology and Laboratory Medicine, UC Davis

^2^Dept. of Surgery, UC Davis

**Key words:** Microbiome, wound, 16S rRNA gene sequencing, bacteria, burn injury, skin graft, and infection.

***Correspondence**

Nam K. Tran, PhD, HCLD (ABB), FAACC

Associate Professor

Director of Clinical Chemistry, Special Chemistry/Toxicology, and Point-of-Care Testing

Dept. of Pathology and Laboratory Medicine

University of California Davis

4400 V St.

Sacramento, CA 95817

Ph: 916-734-8936

Email: [nktran@ucdavis.edu](mailto:nktran@ucdavis.edu)

**SUPPLEMENTAL MATERIAL**


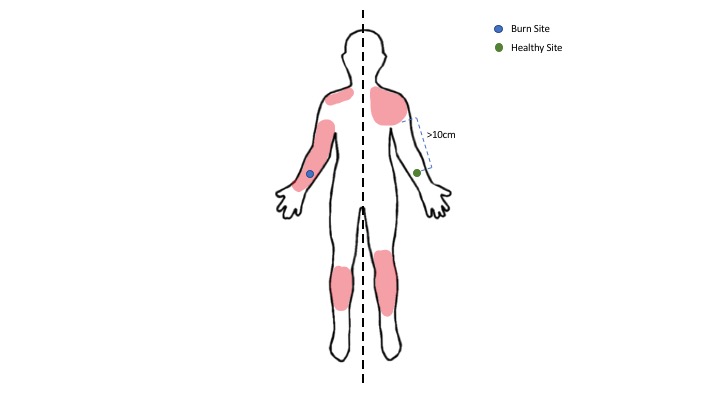


**Figure S1.** Sample Site Determination

This image depicts a hypothetical subject with approximately 10% TBSA anterior burns. For this subject there are several areas of burned tissue that could be selected. To maintain a spared site that is a minimum of 10cm away from injured skin, optimal sampling would be from the right forearm (Burn Site), and the corresponding location on the left forearm (Spared Site). The healthy site is distal enough to not be obstructed by peripheral line placement. This figure was generated using the software Inkscape.

**Figure S2.** Bioinformatics Flow Chart

This figure was generated using Microsoft Powerpoint.


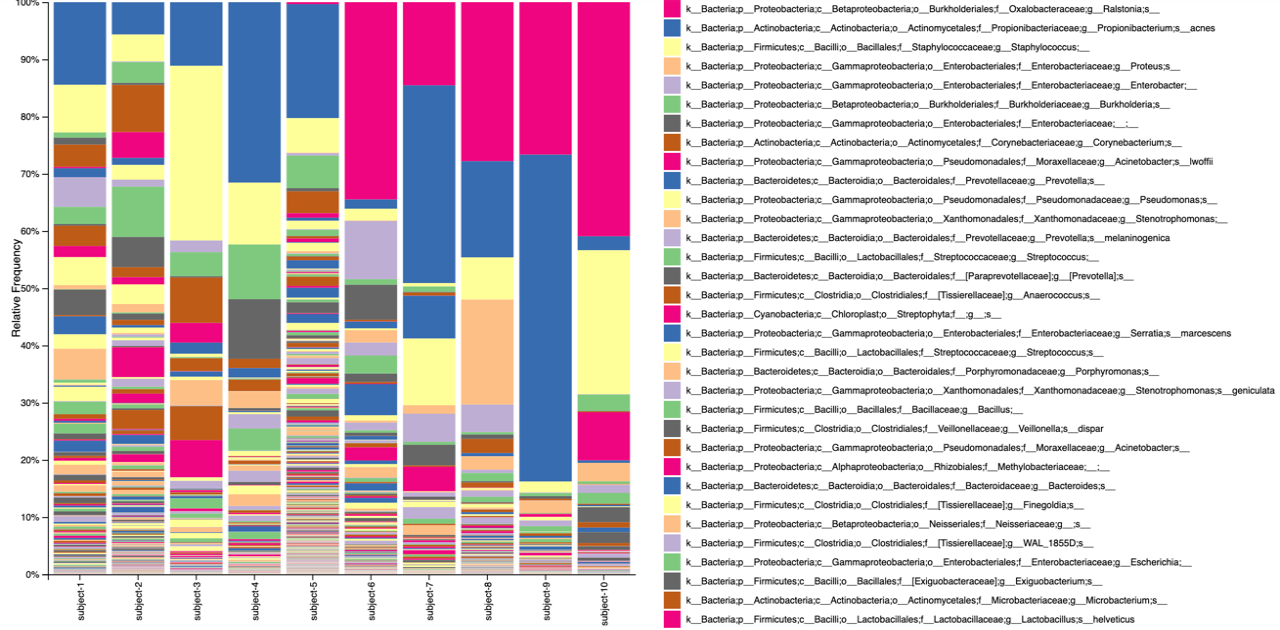


**Figure S3.** Taxonomy of Burn Patient Spared Skin.

Introduction of Ralstonia contaminant at Subject 6 (pink) following the start of a new extraction kit. Spared skin sample site has low microbial abundance and contaminants can predominate when comparing relative abundance. This figure was generated using QIIME2.


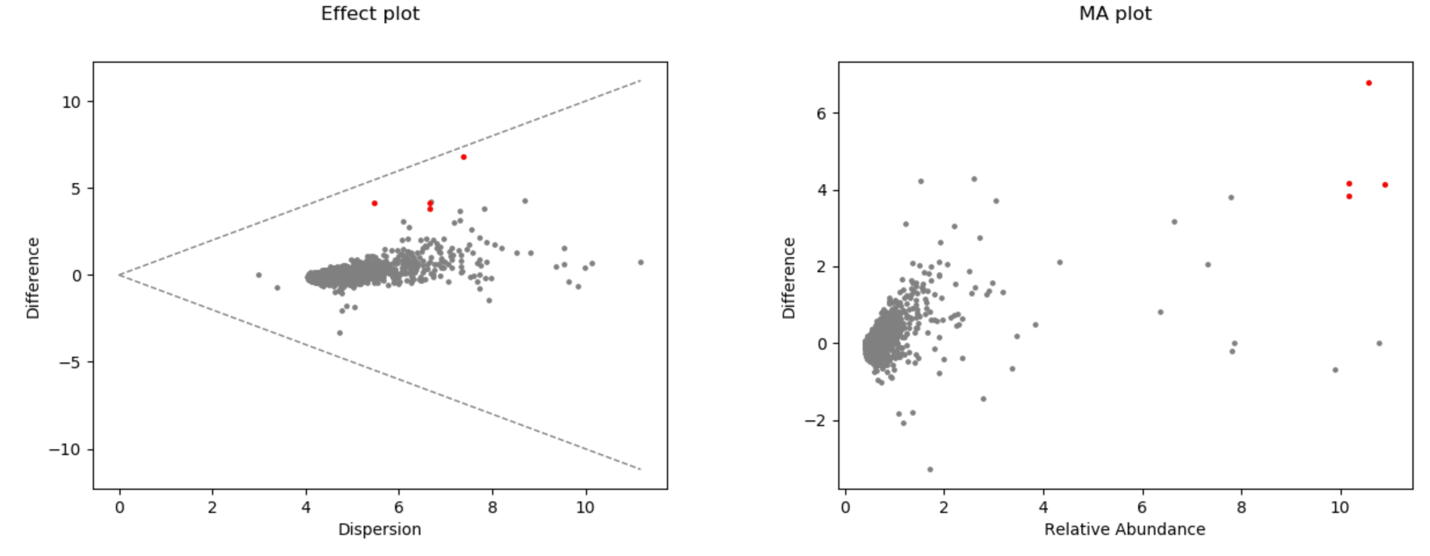

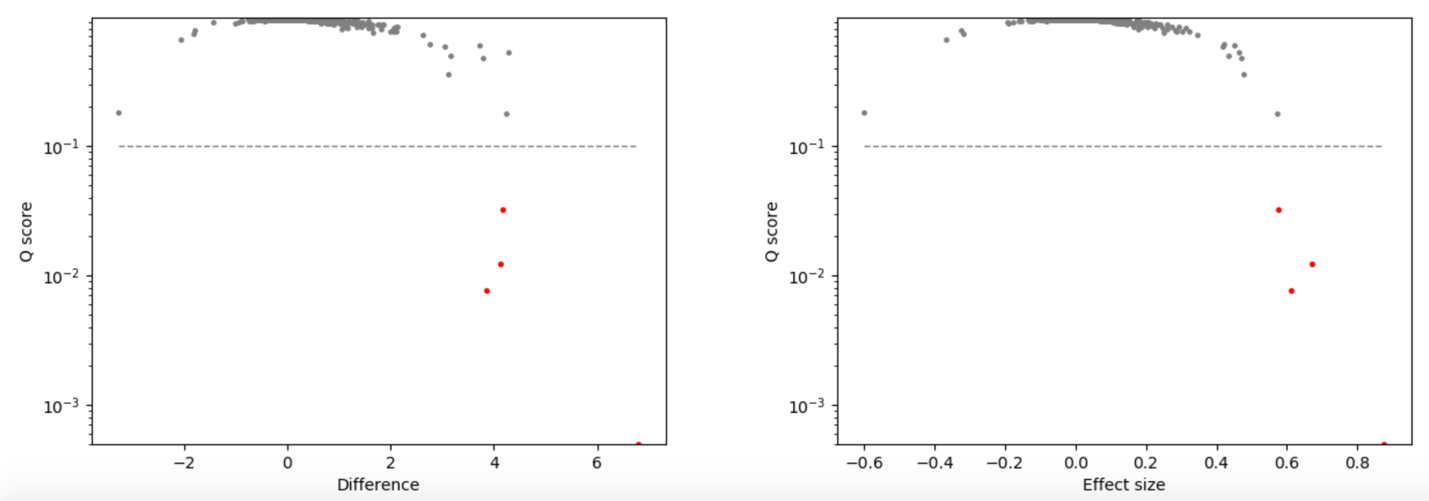


**Figure S4.** ALDEx2 Differential Abundance Between Burn Wound and Spared Skin

This figure was generated using the software Inkscape and visualizations generated by QIIME2.

**Figure S5.** Taxonomic summary of the top 10 genera sequenced from the sum of perianal and rectal samples separated by diet type at the time of collection. This figure was generated using qiime2R.


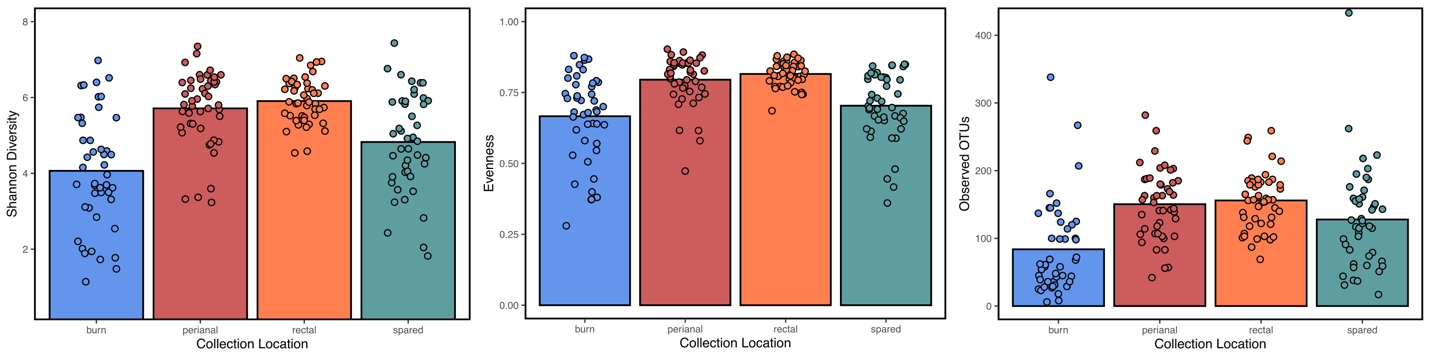


**Figure S6.** Measures of Alpha Diversity

This figure was generated using the software Inkscape and visualizations generated by qiime2R.


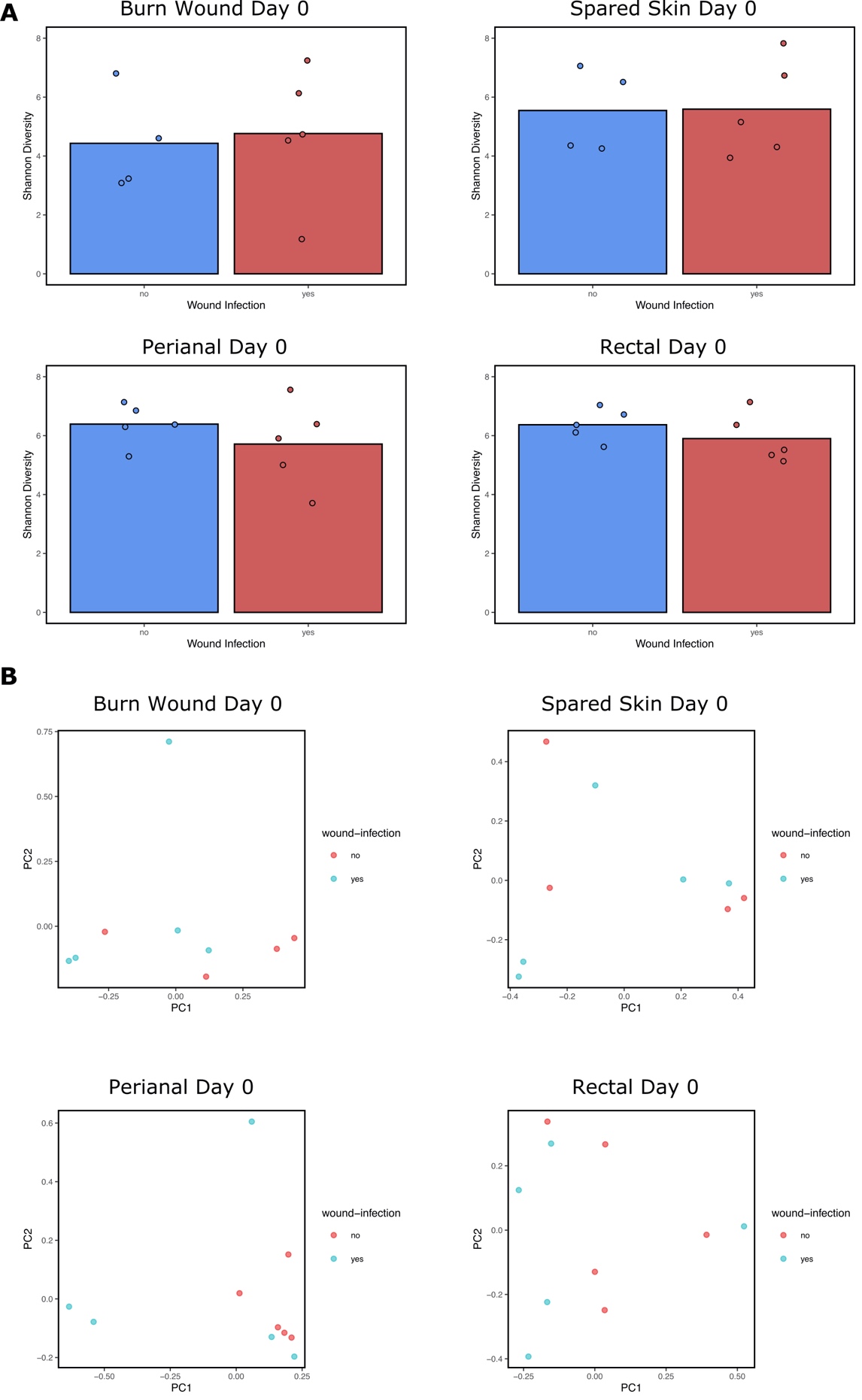


**Figure S7.** Alpha and Beta Diversity of Samples Collected on Admission by Wound Infection Status

(**S7a**) Bar plots of Shannon Diversity by wound infection development during the study and collection location. All collection locations are not significant by Kruskal Wallis comparison between groups: Burn (*P=0.624*), Spared (*P=1.00)*, Perianal (*P=0.251*), and Rectal (*P=0.465*). (**S7b**) Bray Curtis dissimilarity PCOA plot of all burn patient swabs displayed by subject and collection day. In the burn wound plot, the first and second principal components (PC1 and PC2) are shown, explaining 22.66% and 18.41% of the variance, respectively. In the spared skin plot, PC1 and PC2 explain 29.31% and 16.7% of the variance, respectively. In the perianal plot, PC1 and PC2 explain 29.82% and 16.2% of the variance, respectively. In the rectal plot, PC1 and PC2 explain 22.51% and 19.74% of the variance, respectively. All collection locations are not significant by PERMANOVA: Burn (*P=0.440*), Spared (*P=0.492*), Perianal (*P=0.131*), and Rectal (*P=0.403*). This figure was generated using the software Inkscape and visualizations generated by qiime2R.
